# A whole-food, plant-based intensive lifestyle intervention improves glycaemic control and reduces medications in individuals with type 2 diabetes: a randomised controlled trial

**DOI:** 10.1007/s00125-024-06272-8

**Published:** 2024-09-21

**Authors:** Cody J. Hanick, Courtney M. Peterson, Brenda C. Davis, Joan Sabaté, John H. Kelly

**Affiliations:** 1 Department of Nutrition Sciences, University of Alabama at Birmingham, Birmingham, AL USA; 2 Brenda Davis Nutrition Consultation Services, Kelowna, BC Canada; 3 School of Public Health, Center for Nutrition, Lifestyle, and Disease Prevention, Loma Linda University, Loma Linda, CA USA; 4 Department of Preventive Medicine, School of Medicine, Loma Linda University, Loma Linda, CA USA; 5 Lifestyle Health Education Inc., Rocky Mount, VA USA

**Keywords:** Cardiovascular disease, Diabetes remission, Diet, Dietary intervention, Glycaemic control, Lifestyle intervention, Nutrition, Plant-based diet, Randomised controlled trial, Type 2 diabetes

## Abstract

**Aims/hypothesis:**

We conducted the largest and longest clinical trial comparing a whole-food, plant-based intervention with standard medical care (SMC) in individuals with type 2 diabetes.

**Methods:**

We randomised (parallel-arm; computerised 1:1 randomisation ratio) 169 adults aged 18–75 years with type 2 diabetes in the Marshall Islands to an intensive whole-food, plant-based intervention with moderate exercise (PB+Ex) or SMC for 24 weeks. The PB+Ex intervention included 12 weeks of meals, exercise sessions and group classes. Primary outcomes were glycaemic control (HbA_1c_, glucose, insulin and HOMA-IR) and glucose-lowering medication use. Secondary outcomes included lipids, blood pressure, heart rate and C-reactive protein. Only lab analysts were blinded.

**Results:**

Compared with SMC (*n*=90 randomised; *n*=70 analysed), the PB+Ex (*n*=79 randomised; *n*=66 analysed) intervention decreased HbA_1c_ by an additional 14 mmol/mol (1.3%) at week 12 (−22 vs −7 mmol/mol [−2.0% vs −0.7%]; *p*<0.0001) and 8 mmol/mol (0.7%) at week 24 (−16 vs −8 mmol/mol [−1.4% vs −0.7%]; *p*=0.01). Concomitantly, 63% of medicated PB+Ex participants reduced their glucose-lowering medications (vs 24%; *p*=0.006), and 23% of PB+Ex participants with a baseline HbA_1c_ <75 mmol/mol (<9%) achieved remission. Additionally, the PB+Ex intervention reduced weight (−2.7 kg; *p*<0.0001), C-reactive protein (−11 nmol/l; *p*=0.005) and cardiovascular medication use compared with SMC. At intermediate timepoints, it improved glucose, insulin, HOMA-IR, cholesterol, triglycerides and heart rate, but not at week 24.

**Conclusions/interpretation:**

A whole-food, plant-based lifestyle intervention was more effective for improving glycaemic control than SMC. It also reduced the need for diabetes and cardiovascular medications and induced diabetes remission in some participants. Therefore, it is an effective, evidence-based lifestyle option for individuals with type 2 diabetes.

**Trial registration:**

ClinicalTrials.gov NCT03862963

**Funding:**

This research was funded by the Department of the Army (W81XWH-05-1-0547). CJH received support through a National Institutes of Health Predoctoral T32 Obesity Fellowship (T32 HL105349).

**Graphical Abstract:**

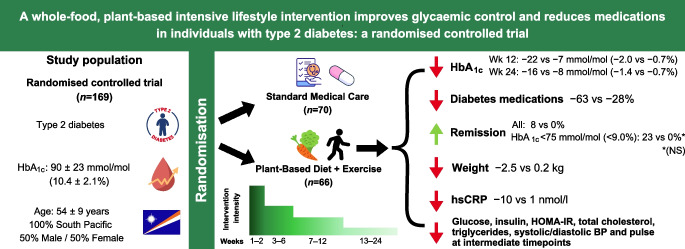

**Supplementary Information:**

The online version contains peer-reviewed but unedited supplementary material available at 10.1007/s00125-024-06272-8.


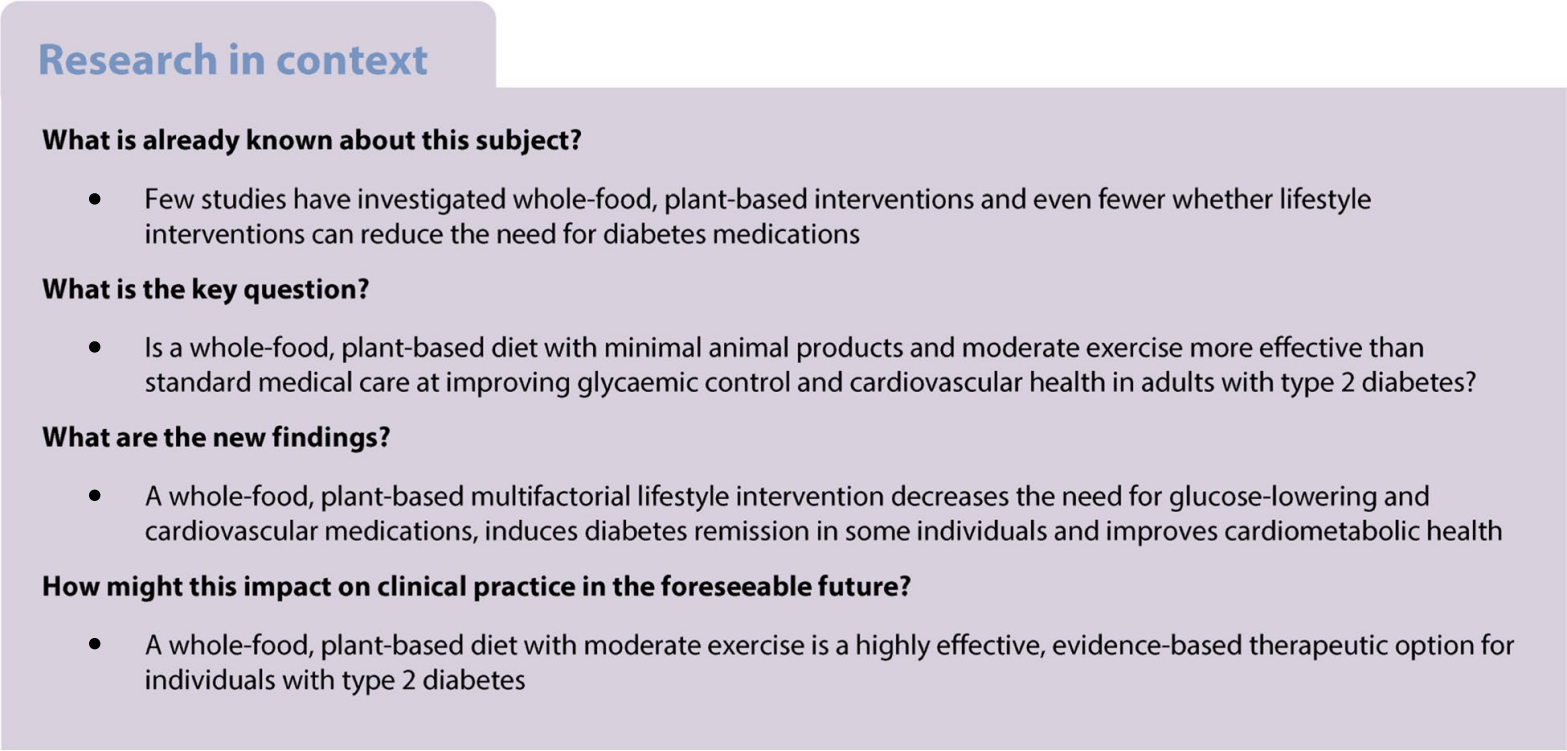


## Introduction

Diabetes is a pressing global health issue, affecting 10.5% of adults worldwide [1]. Although the causes of type 2 diabetes are complex, diet is a leading risk factor. Data from the Global Burden of Disease study suggest that a poor diet (low in vegetables, fruit, whole grains and fibre, and high in sugar-sweetened beverages, processed meat and red meat) is the second leading risk factor for diabetes, behind only a high BMI [2].

Increasingly, data suggest that a healthy diet can not only prevent type 2 diabetes but may also treat and reverse it. The Diabetes Remission Clinical Trial (DiRECT) found that a diet very low in energy (very-low-calorie diet [VLCD]) decreased HbA_1c_ by 10 mmol/mol (0.9%), and 46% of participants with early-stage type 2 diabetes went into remission vs only 4% receiving standard care [3]. However, VLCDs fail to improve HbA_1c_ in up to 40% of individuals, they are difficult to adhere to and only a minority of individuals are willing to try them [3–5]. Moreover, VLCDs pose several health risks, including headaches, dizziness, muscle cramps and bone loss [3].

Therefore, improving diet quality may be a better approach for treating type 2 diabetes. Of these approaches, low-carbohydrate diets, particularly ketogenic diets, are the most well-studied. Clinical trials suggest ketogenic diets lower HbA_1c_ [6], reduce diabetes medications [6] and induce diabetes remission in some individuals [7]. However, low-carbohydrate diets are associated with increased mortality risk [8] and can actually impair glucose tolerance [9–11] and increase cholesterol and inflammation [11, 12].

An alternative, potentially more promising, approach is whole-food, plant-based (WFPB) diets, which are predominated by whole foods such as vegetables, fruits, legumes, whole grains, nuts and seeds. WFPB diets include not only vegetarian and vegan diets but also other plant-predominant diets that incorporate meat and seafood. Clinical trials in adults with type 2 diabetes have found that WFPB diets lower HbA_1c_ [13–15], reduce the need for diabetes medications [16–20] and allow some individuals to wean off all medications [16, 18, 19]. For example, one small study found that 55% of individuals with insulin-dependent type 2 diabetes no longer needed exogenous insulin after only 16 days on a WFPB diet [19]. In addition, WFPB diets can reduce body weight without deliberate energy restriction [21, 22], have fewer side effects than VLCDs and ketogenic diets and may also have better adherence and acceptability [23, 24].

Although promising, most studies examining WFPB diets and diabetes are small and short-term. Thus, we conducted the largest and longest randomised controlled trial to compare a WFPB intervention vs standard medical care (SMC) in participants with type 2 diabetes. We conducted the clinical trial in the Republic of the Marshall Islands (RMI), which has the seventh-highest diabetes prevalence globally [25]. The country’s high prevalence has been partially attributed to its increased reliance on imported foods, including white rice, refined flour, sugar-sweetened beverages and canned meats [26–31], which provides an excellent milieu to test whether improving diet quality can treat type 2 diabetes. To mirror other intensive lifestyle interventions, we paired a WFPB diet with moderate exercise and hypothesised that the combination would be more effective than SMC for improving glycaemic control and cardiovascular health and would reduce the need for glucose-lowering medications.

## Methods

### Study design

We conducted a 24 week parallel-arm, randomised controlled trial comparing a whole-food, plant-based intervention with moderate exercise (PB+Ex) with SMC in adults with type 2 diabetes. The study was approved by the institutional review board at Loma Linda University (protocol no. 59105) and an ad hoc institutional review board assembled by the RMI Minister of Health.

### Study population

We enrolled adults with type 2 diabetes aged 18–75 years. Participants either had an HbA_1c_ ≥64 mmol/mol (≥8.0%) or were diagnosed with type 2 diabetes and taking glucose-lowering medication. Exclusion criteria included heart disease, changes in glucose-lowering medications in the past 3 months and physical or medical conditions that hinder participation. Enrolment was generally representative of Marshallese adults with type 2 diabetes (see the electronic supplementary material [ESM]). Participants provided written informed consent before participating, and demographic variables including biological sex were collected by self-report. Participants were enrolled in five cohorts and randomised in a 1:1 ratio generated by Microsoft Excel. Participants were randomised a few days before baseline data collection to give them time to make accommodations to attend weekly classes. Further details on recruitment, randomisation, the lifestyle intervention and the methods are provided in the protocol manuscript [32].

### Standard care

The control group was treated using glucose-lowering pharmacotherapy, according to SMC in the RMI. They were instructed to maintain their current diet and physical activity levels.

### PB+Ex intervention

The PB+Ex intervention is described in detail in the protocol manuscript [32]. In brief, the PB+Ex group was instructed to eat a WFPB diet permitting minimal animal products and to exercise 30–60 min/day for 24 weeks. During weeks 1–12, the PB+Ex group received prepared meals, attended group exercise sessions and received group instruction on eating healthfully, cooking, exercising and managing stress (see ESM Table 1 for a list of class topics). The intervention was culturally tailored and developed in partnership with the Marshallese government and local diabetes clinics and included Marshallese staff and popular foods (see the ESM for a detailed description of the cultural adaptation and positionality statements). The intensity of support progressively decreased, with participants attending 15–21 h/week of group classes in weeks 1–2, 8–10 h/week in weeks 3–6 and 4–5 h/week in weeks 7–12. During weeks 13–24 (the follow-up phase), participants were instructed to follow the intervention on their own.

#### WFPB diet

The prescribed diet was high in fibre (35 g/ 4184 kJ), low in fat (20–25% of energy; saturated fat <7% of energy), moderate in protein (10–15% of energy) and low in sodium (<2400 mg/day). During weeks 1–2 (the intensive phase), PB+Ex participants received 12 prepared meals/week and were instructed to consume no animal products and minimal ground grains and refined carbohydrates. Thereafter, participants received 2 meals/week during weeks 3–6 and 1 meal/week during weeks 7–12. During weeks 3–12, participants could consume small amounts of animal foods, oils, fat-rich foods and processed foods, following a four-tiered food classification system [33]. Specifically, they were instructed to consume 75–100% of energy from whole, unprocessed plant foods (tier 1), such as vegetables, legumes, whole grains and fruit. The remainder of their diet could include ≤25% lightly processed foods (tier 2), ≤10% moderately processed foods and moderate-fat animal products (tier 3) and ≤5% heavily processed foods and high-fat animal products (tier 4).

#### Exercise

The PB+Ex group was instructed to do moderate-intensity aerobic and resistance exercise 60 min/day during weeks 1–2 and 30–60 min/day during weeks 3–24. During weeks 1–2, participants attended 1 h group exercise classes 4 days/week. Thereafter, they attended group exercise classes twice a week during weeks 3–6 and once a week during weeks 7–12. Participants were also counselled to walk 10–20 min before breakfast and after lunch and dinner.

#### Cohort differences

To increase intervention intensity, participants in cohorts 3–5 of the PB+Ex group repeated weeks 1 and 2 during weeks 4 and 6. This included a repeat of educational sessions, meals provided and exercise classes.

### Study outcomes

Outcomes were assessed at weeks 0, 2, 6, 12 and 24. The primary outcome was glycaemic control, measured by HbA_1c_, fasting glucose, fasting insulin, HOMA-IR and diabetes medication use. Secondary endpoints were cardiovascular risk factors, including body weight, waist circumference, lipids, systolic blood pressure (SBP), diastolic blood pressure (DBP), resting heart rate, high-sensitivity C-reactive protein (hsCRP) and cardiovascular medication use. Only lab analysts performing the serum assays were blinded.

### Serum chemistry

HbA_1c_, glucose, insulin, total cholesterol, HDL-cholesterol, triglycerides and hsCRP were analysed blinded at the Clinical Laboratory Improvement Amendments-approved laboratory in the Ministry of Health’s Hospital, while LDL-cholesterol was calculated using the Friedewald equation. Triglyceride values >4.52 mmol/l (>400 mg/dl) were Winsorised to minimise the effect of outliers on the analyses. LDL-cholesterol values were treated as missing whenever triglyceride values exceeded 4.52 mmol/l. hsCRP values ≥95 mmol/l (≥10 mg/l) were considered indicative of acute infection and treated as missing.

### Medication use

Primary care physicians and/or the Diabetes Wellness Clinic’s clinicians adjusted participants’ medications based on glucometer and/or serum glucose values. PB+Ex participants on insulin were monitored daily with glucometers and instructed to reduce insulin doses when glucose fell to <3.9 mmol/l (<70 mg/dl) or hypoglycaemic symptoms manifested. For SMC participants on insulin, their physicians were responsible for adjusting their medication doses. Diabetes medication use was quantified using the medication effect score (MES) [25], which estimates the HbA_1c_ reduction expected from all glucose-lowering pharmacotherapy [34]. Diabetes remission was defined as achieving HbA_1c_ <48 mmol/mol (<6.5%) after not using glucose-lowering medications for at least 3 months.

### Statistical power

The required sample size was estimated using the variances observed in HbA_1c_ and glucose in cohorts 1–2. A sample size of *n*=120 was needed to have 80% power to detect a 1.1 mmol/l (20 mg/dl) difference in glucose and an 11 mmol/mol (1.0%) difference in HbA_1c_, given α=0.05.

### Statistical analyses

Analyses were performed with two-sided tests and α=0.05, primarily using SAS (version 9.4; SAS Institute; Cary, NC, USA). Baseline data were compared using independent samples *t* tests or the Mann–Whitney *U* test if neither raw nor transformed values were normal. The main analysis was intention-to-treat. Continuous data were analysed using linear mixed models, adjusting for baseline values, sex and/or cohort whenever statistically merited. Categorical data were analysed using Fisher’s exact test. Missing medication doses were singly imputed whenever a missing dose was flanked by two timepoints with identical doses and were otherwise treated as missing. When HbA_1c_ values were missing, remission status was singly imputed by assuming HbA_1c_ values changed by no more than 16 mmol/mol (1.5%) between weeks 0 and 2 and by no more than 33 mmol/mol (3.0%) between each subsequent pair of timepoints. Insulin and HOMA-IR were analysed only in participants not on insulin, while MES was analysed only in those taking glucose-lowering medication. The proportion of participants who decreased their medication doses was calculated in the subgroup of participants on the medication(s) at baseline. Lastly, to calculate diabetes remission and decreases in medication doses, we included everyone with sufficient data at either week 12, week 24 or both timepoints, in order to increase the sample size and improve accuracy in the estimated proportions.

## Results

### Participants

As shown in ESM Fig. 1, we screened 530 people. Of these, 361 did not meet the eligibility criteria, primarily due to HbA_1c_ being out of range and secondarily due to cardiovascular pathologies, such as angina. We randomised 169 participants (SMC: *n*=90; PB+Ex: *n*=79), but 31 withdrew before baseline data were collected. Seventy-two participants received the SMC intervention, while 66 received the PB+Ex intervention. Twenty-eight participants (20%) were lost to follow-up at week 24, and retention was similar between groups (*p*=1.00). Two completers were excluded from the analyses after we later discovered that they no longer had diabetes at baseline. There were no adverse events related to the protocol. Participant characteristics are summarised in Table 1. All participants were of Pacific Islander descent, and 50% were female. Participants had a mean age (±SD) of 54 ± 9 years, a BMI of 29.8 ± 4.9 kg/m^2^, an HbA_1c_ of 90 ± 23 mmol/mol (10.4 ± 2.1%) and a fasting glucose of 12.9 ± 4.2 mmol/l, indicating high rates of uncontrolled type 2 diabetes. Sixty-one per cent of participants used ≥1 glucose-lowering agent, and metformin and sulfonylureas were each used by 40% of participants. Only 9% of participants were on insulin. Thirty-two per cent of participants were taking ≥1 cardiovascular medication. All participant characteristics were similar between groups at baseline, except SBP, which was 8 mmHg higher in the PB+Ex group (*p*=0.04). Thus, blood pressure analyses were adjusted for baseline values.

**Table 1:** Baseline characteristics

Characteristic	Total (*n*=136)	SMC (*n*=70)	PB+Ex (*n*=66)	*p*
Demographics				
Age, years	54 ± 9	53 ± 9	55 ± 9	0.22
Female	68 (50)	32 (46)	36 (55)	0.39
Anthropometrics				
Weight, kg	77.6 ± 14.2	76.2 ± 14.6	79.0 ± 13.7	0.25
BMI, kg/m^2^	29.8 ± 4.9	29.2 ± 4.9	30.5 ± 4.8	0.07
Waist circumference, cm	97.7 ± 10.0	96.6 ± 10.0	98.8 ± 10.0	0.19
Glycaemic parameters				
HbA_1c_, mmol/mol	90 ± 23	92 ± 23	88 ± 23	0.35
HbA_1c_, %	10.4 ± 2.1	10.5 ± 2.1	10.2 ± 2.1	0.35
Glucose, mmol/l	12.9 ± 4.2	13.1 ± 4.3	12.7 ± 4.1	0.58
Insulin, pmol/l	60.5 ± 49.7	58.0 ± 45.5	63.4 ± 54.2	0.55
HOMA-IR	5.63 ± 5.52	5.61 ± 6.31	5.64 ± 4.54	0.56
MES, mmol/mol	12 ± 10	10 ± 8	13 ± 11	0.39
MES, %	1.1 ± 0.9	0.9 ± 0.7	1.2 ± 1.0	0.39
No. diabetes medications	1.0 ± 1.0	0.9 ± 0.9	1.1 ± 1.0	0.15
Diabetes medication use	83 (61)	39 (56)	44 (67)	0.22
Metformin	55 (40)	25 (36)	30 (45)	0.30
Sulfonylureas	54 (40)	26 (37)	28 (42)	0.60
Insulin	12 (9)	6 (9)	6 (9)	1.00
Thiazolidinedione	1 (1)	1 (1)	0 (0)	1.00
Cardiovascular risk factors				
SBP, mmHg	121 ± 22	118 ± 21	125 ± 22	0.04
DBP, mmHg	73 ± 11	72 ± 11	74 ± 10	0.19
Heart rate, beats/min	78 ± 10	78 ± 9	78 ± 11	0.97
Total cholesterol, mmol/l	4.88 ± 1.07	4.88 ± 0.97	4.88 ± 1.16	0.98
Triglycerides, mmol/l	2.11 ± 1.11	2.04 ± 1.09	2.18 ± 1.14	0.44
LDL-cholesterol, mmol/l	3.02 ± 0.97	3.03 ± 0.91	3.01 ± 1.04	0.91
HDL-cholesterol, mmol/l	0.92 ± 0.24	0.91 ± 0.21	0.94 ± 0.26	0.49
hsCRP, nmol/l	32 ± 20	29 ± 19	34 ± 22	0.22
No. cardiovascular medications	0.6 ± 1.1	0.5 ± 1.0	0.7 ± 1.1	0.38
Cardiovascular medication use	43 (32)	20 (29)	23 (35)	0.46
Lipid-lowering medication	24 (18)	9 (13)	15 (23)	0.18
Antihypertensive medication	20 (15)	10 (14)	10 (15)	1.00
Aspirin	33 (24)	13 (19)	20 (30)	0.16

Data are *n* (%) or mean ± SD unless otherwise noted

### Glycaemic outcomes

**Fig. 1:**
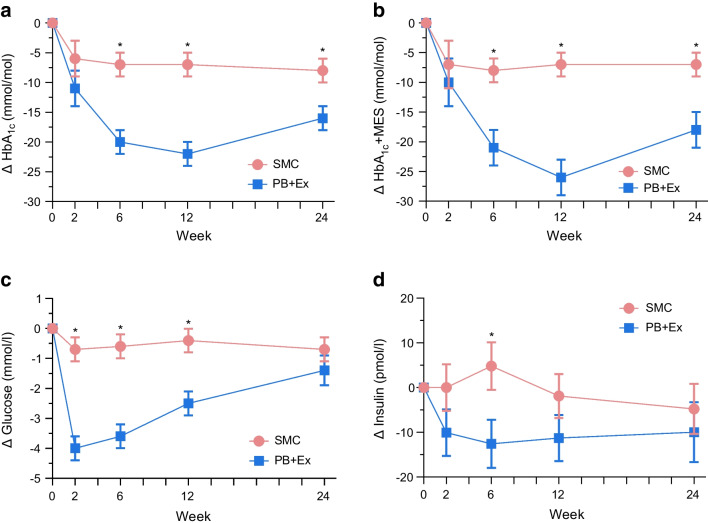
Glycaemic control. The PB+Ex intervention was more effective than SMC at improving (**a**) HbA_1c_ and (**b**) the MES plus HbA_1c_ (HbA_1c_+MES), which measures the true effect on HbA_1c_ if glucose-lowering medication doses had not been changed. The PB+Ex intervention also improved (**c**) fasting glucose at all timepoints except week 24 and (**d**) fasting insulin at week 2 only. Data shown are least-squares means ± SEMs. *p<0.05

ESM Table 2 summarises the results for all cardiometabolic outcomes. Glycaemic outcomes are illustrated in Fig. 1. Compared with SMC, the PB+Ex intervention reduced HbA_1c_ by an additional 13 mmol/mol (1.2%) at week 6 (−20 vs −7 mmol/mol [−1.8% vs −0.6%]; 95% CI −19, −7 mmol/mol [−1.7%, −0.6%]; *p*<0.0001), 14 mmol/mol (1.3%) at week 12 (−22 vs −7 mmol/mol [−2.0% vs −0.7%]; 95% CI −20, −8 mmol/mol [−1.8%, −0.8%]; *p*<0.0001) and 8 mmol/mol (0.7%) at week 24 (−16 vs −8 mmol/mol [−1.4% vs −0.7%]; 95% CI −14, −1 mmol/mol [−1.3%, −0.1%]; *p*=0.01) (Fig. 1a). Since many participants decreased their glucose-lowering medication doses, we used the MES to calculate the true reduction in HbA_1c_ if medication doses had not been adjusted (i.e. HbA_1c_+MES; Fig. 1b). Had medication doses not been changed, the PB+Ex intervention would have reduced HbA_1c_ by an additional 13 mmol/mol (1.2%) at week 6 (−21 vs −8 mmol/mol [−1.9% vs −0.8%]; 95% CI −20, −6 mmol/mol [−1.8%, −0.5%]; *p*=0.0004), 19 mmol/mol (1.7%) at week 12 (−26 vs −7 mmol/mol [−2.4% vs −0.6%]; 95% CI −26, −12 mmol/mol [−2.4%, −1.1%]; *p*<0.0001) and 11 mmol/mol (1.0%) at week 24 (−18 vs −7 mmol/mol [−1.6% vs −0.6%]; 95% CI −18, −3 mmol/mol [−1.7%, −0.3%]; *p*=0.005). The PB+Ex intervention also reduced glucose by an additional 3.2 mmol/l at week 2 (95% CI −4.4, −2.0 mmol/l; *p*<0.0001), 3.0 mmol/l at week 6 (95% CI −4.2, −1.8 mmol/l; *p*<0.0001) and 2.1 mmol/l at week 12 (95% CI −3.3, −0.9 mmol/l; *p*=0.0007) relative to SMC, but not at week 24 (*p*=0.24) (Fig. 1c). Among participants not taking insulin (*n*=124), the PB+Ex intervention reduced fasting insulin at week 6 by 17.4 pmol/l (−12.6 vs −4.8 pmol/l; 95% CI −32.4, −2.5 pmol/l; *p*=0.02) but did not affect insulin at any other timepoint (*p*≥0.17; Fig. 1d). The PB+Ex intervention also reduced HOMA-IR at week 2 (−1.74; 95% CI −3.37, −0.11; *p*=0.04), week 6 (−2.98; 95% CI −4.61, −1.35; *p*=0.0004) and week 12 (−1.74; 95% CI −3.25, −0.23; *p*=0.02), but not at week 24 (*p*=0.53) (ESM Fig. 2b).

### Diabetes remission and medication use

**Fig. 2:**
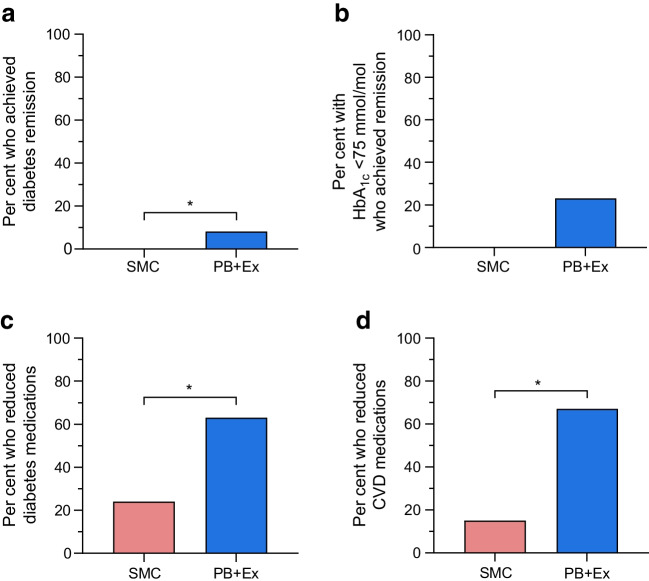
Diabetes remission and medication use. (**a**) PB+Ex was more effective than SMC at inducing diabetes remission. (**b**) About one-quarter of PB+Ex participants with a baseline HbA_1c_ <75 mmol/mol (<9%) achieved remission. The PB+Ex group also significantly reduced their doses of (**c**) diabetes medications and (**d**) cardiovascular medications. Data shown are proportions (%). *p<0.05

Diabetes remission rates and medication use are shown in Fig. 2. Eight per cent (*n*=5) of PB+Ex participants achieved diabetes remission vs 0% in the SMC group (*n*=127; *p*=0.02; Fig. 2a). All PB+Ex participants who achieved remission had a baseline HbA_1c_ <75 mmol/mol (<9.0%). In a post hoc analysis, the PB+Ex intervention induced remission in 23% of participants with a baseline HbA_1c_ <75 mmol/mol (<9.0%), although this was not statistically different from the SMC group (vs 0%; *n*=36; *p*=0.13; Fig. 2b). In addition, 63% of PB+Ex participants reduced their baseline dose of glucose-lowering medications vs only 24% of SMC participants (*n*=56; *p*=0.006). This was mirrored by statistically significant decreases in MES at all timepoints, including at week 12 (−6 mmol/mol [−0.5%]; 95% CI −8, −3 mmol/mol; *p*=0.0002) and week 24 (−9 mmol/mol [−0.7%]; 95% CI −12, −5 mmol/mol; *p*<0.0001) (*n*=91; ESM Fig. 2a). Lastly, 67% of PB+Ex participants reduced their dose of one or more cardiovascular medications vs only 15% of the SMC group (*n*=19; *p*=0.046).

### Body weight and cardiovascular disease risk factors

**Fig. 3:**
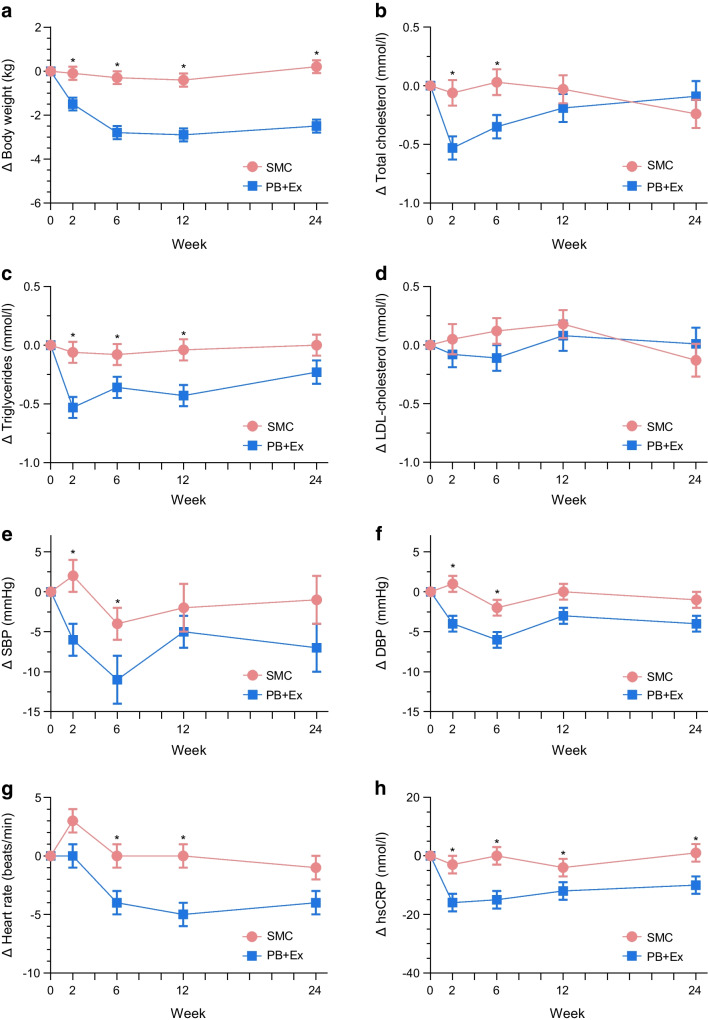
Cardiovascular disease risk factors. The PB+Ex intervention was more effective than SMC at lowering (**a**) body weight and (**h**) hsCRP. The PB+Ex intervention improved (**b**) total cholesterol, (**c**) triglycerides, (**e**) SBP, (**f**) DBP and (**g**) heart rate at intermediate timepoints but not at week 24. There were no differences in (**d**) LDL-cholesterol. Data shown are least-squares means ± SEMs. *p<0.05

Figure 3 shows the effects on body weight and cardiovascular risk factors. Relative to SMC, the PB+Ex intervention modestly reduced body weight at all timepoints, including week 2 (−1.4 kg; 95% CI −2.2, −0.6 kg; *p*=0.001), week 6 (−2.6 kg; 95% CI −3.4, −1.7 kg; *p*<0.0001), week 12 (−2.5 kg; 95% CI −3.4, −1.6 kg; *p*<0.0001; Fig. 3a) and week 24 (−2.7 kg; 95% CI −3.6, −1.8 kg; *p*<0.0001). Similarly, waist circumference was significantly reduced in the PB+Ex group at weeks 6 (−1.8 cm; 95% CI −3.6, 0.0 cm; *p*=0.04), 12 (−1.9 cm; 95% CI −3.7, −0.1 cm; *p*=0.04) and 24 (−3.8 cm; 95% CI −5.8, −1.8 cm; *p*=0.0002), but not at week 2 (*p*=0.34; ESM Fig. 2c). The PB+Ex intervention also reduced total cholesterol by an additional 0.47 mmol/l at week 2 (95% CI −0.76, −0.19 mmol/l; *p*=0.001) and 0.38 mmol/l at week 6 (95% CI −0.67, −0.08 mmol/l; *p*=0.01), but not at week 12 or week 24 (*p*≥0.34; Fig. 3b). This was driven by large decreases in triglycerides at week 2 (−0.47 mmol/l; 95% CI −0.72, −0.23 mmol/l; *p*=0.0002), week 6 (−0.28 mmol/l; 95% CI −0.53, −0.04 mmol/l; *p*=0.02) and week 12 (−0.38 mmol/l; 95% CI −0.63, −0.13 mmol/l; *p*=0.003), but not at week 24 (*p*=0.09; Fig. 3c). There were no differences in LDL-cholesterol (*p*≥0.13; Fig. 3d) or HDL-cholesterol (*p*≥0.09; ESM Fig. 1d) at any timepoint. In addition, the PB+Ex intervention reduced SBP by 8 mmHg at both week 2 (95% CI −14, −1 mmHg; *p*=0.02) and week 6 (95% CI −14, −1 mmHg; *p*=0.03), but not at week 12 or week 24 (*p*≥0.13; Fig. 3e). Similarly, the PB+Ex intervention reduced DBP by 5 mmHg at week 2 (95% CI −9, −2 mmHg; *p*=0.003) and 4 mmHg at week 6 (95% CI −8, 0 mmHg; *p*=0.03), but not at week 12 or week 24 (*p*≥0.08; Fig. 3f). Relative to SMC, the PB+Ex intervention also reduced heart rate by 4 beats/min at week 6 (95% CI −8, −1 beats/min; *p*=0.02) and 5 beats/min at week 12 (95% CI −8, −1 beats/min; *p*=0.02), but not at week 2 or week 24 (*p*≥0.10; Fig. 3g). Lastly, the PB+Ex intervention more effectively lowered hsCRP at all timepoints, including week 2 (−14 nmol/l; 95% CI −21, −6 nmol/l; *p*=0.0003), week 6 (−14 nmol/l; 95% CI −22, −7 nmol/l; *p*=0.0003), week 12 (−9 nmol/l; 95% CI −16, −1 nmol/l; *p*=0.02) and week 24 (−11 nmol/l; 95% CI −19, −4 nmol/l; *p*=0.005; Fig. 3h).

## Discussion

We conducted the largest and longest randomised controlled trial to compare a WFPB intervention vs SMC in individuals with type 2 diabetes. We implemented a lifestyle intervention involving a WFPB diet including limited animal products and moderate exercise, which progressively decreased in intensity. The PB+Ex intervention was superior to SMC for improving HbA_1c_, hsCRP, weight and waist circumference. The PB+Ex intervention also reduced the need for diabetes and cardiovascular medications and induced type 2 diabetes remission in some participants. At interim timepoints, the PB+Ex intervention improved nearly every cardiometabolic endpoint, although these differences attenuated as the intervention intensity decreased.

The PB+Ex intervention was far more effective at improving glycaemic control than SMC centred on medication management: it decreased HbA_1c_ by an additional 14 mmol/mol (1.3%) at week 12 and 8 mmol/mol (0.7%) at week 24. Importantly, the ‘true’ effect on HbA_1c_ levels was even larger than this because the SMC group increased their dose of glucose-lowering medications by the equivalent of 360 mg/day of metformin at week 24, whereas the PB+Ex group reduced their dose by 820 mg/day (a between-group difference of 1180 mg/day) [34]. After adjusting for medication changes, the PB+Ex intervention lowered HbA_1c_ by an additional 19 mmol/mol (1.7%) at week 12 and 11 mmol/mol (1.0%) at week 24 relative to SMC. Such a large improvement in HbA_1c_ levels could dramatically reduce the risks of comorbidities, particularly myocardial infarction and microvascular complications, and profoundly improve clinical management of type 2 diabetes. Interestingly, much of the glycaemic improvement occurred in the first 2 weeks of the study before any substantial weight loss occurred. The PB+Ex intervention decreased fasting glucose by a dramatic 4.0 mmol/l relative to baseline within only 2 weeks. This suggests that the improvements were due to changes in diet quality and/or physical activity rather than weight loss. For comparison, the glycaemic improvements we observed were much larger than the 7–11 mmol/mol (0.6–1.1%) reduction in HbA_1c_ reported in other clinical trials on plant-based diets [17, 35, 36] or the 3–4 mmol/mol (0.3–0.4%) reported in meta-analyses [13, 37]. Plus, most studies on plant-based interventions report no improvements in fasting glucose or insulin [17, 35, 36, 38, 39]. The larger effects we observed may be due to the very high amounts of whole foods, the addition of moderate exercise, the provision of prepared meals and intensive instruction, participants having higher baseline HbA_1c_ values than in the USA [27] and/or participants having a lower quality diet at baseline [28–31, 40, 41]. A complex interplay of socioeconomic, geopolitical, and cultural factors involving limited arable land, displacement, a remote location, unemployment, and poverty have decreased access to nutritious foods [29, 42].

The PB+Ex intervention also reduced the need for glucose-lowering and cardiovascular medications in roughly two-thirds of PB+Ex participants. Our intervention induced a greater reduction in medication use than in any other clinical trial on plant-based diets [15, 17, 35, 36, 38, 39, 43]. The PB+Ex intervention also induced type 2 diabetes remission in 8% of all participants and 23% of those with a baseline HbA_1c_ <75 mmol/mol (<9.0%). The participants who went into remission lost modest or no weight (~0–6 kg), and one even gained weight. For comparison, in the DiRECT trial, where participants’ mean baseline HbA_1c_ was 61 mmol/mol (7.7%), only 7% of participants who lost 0–5 kg went into remission [3]. This important finding suggests that type 2 diabetes remission is possible through improving diet quality and/or increasing physical activity, even if individuals do not lose weight.

We also investigated the effects of a PB+Ex intervention on cardiovascular risk factors. The PB+Ex intervention reduced total cholesterol and triglycerides at intermediate timepoints but did not affect LDL-cholesterol. It is unclear why we found a dramatic reduction in triglycerides but not LDL-cholesterol, as this conflicts with a meta-analysis reporting that plant-based diets lower total and LDL-cholesterol but do not affect triglycerides [44]. The PB+Ex intervention also decreased SBP by 8 mmHg at both week 2 and week 6 and DBP by 5 and 4 mmHg at weeks 2 and 6, respectively. However, the effects vanished as the intervention intensity waned. The effects we observed at intermediate time points were larger than those reported in a meta-analysis of plant-based diets, which reported 3 and 2 mmHg improvements in SBP and DBP, respectively [45]. The PB+Ex intervention also decreased heart rate by 4 and 5 beats/min at weeks 6 and 12, respectively, compared with the SMC, although the effect lost significance by week 24 (*p*=0.10). These intermediate improvements in triglycerides, blood pressure and heart rate may partially explain why plant-based diets are associated with lower cardiovascular disease incidence and mortality risk [46]. Finally, the PB+Ex intervention reduced hsCRP at all timepoints, indicating the intervention decreased inflammation. Notably, biological sex was not a statistically significant covariate for any cardiometabolic outcome, suggesting no differences between males and females.

The strengths of this study include the innovative diet approach, large sample size, long duration, high male representation (50%), high-priority population, the cultural adaptation and using a lifestyle intervention that progressively decreased in intensity. The latter factor provided us with a unique opportunity to test different ‘doses’ of the PB+Ex intervention. Interestingly, during the most intensive phase, participants experienced robust improvements in nearly every cardiometabolic endpoint, although the effects for fasting glucose, insulin, triglycerides, heart rate and blood pressure waned as intervention intensity decreased. This suggests that a WFPB diet with moderate exercise can improve most cardiometabolic risk factors, but the effects depend on the intervention intensity and/or level of adherence. Limitations of this study include that no adherence data or diet records were collected; participants were randomised 3–5 days prior to baseline testing; some SMC participants adopted elements of the PB+Ex lifestyle intervention (which likely diluted estimates of the true treatment effects); no Marshallese citizens assisted with study design, data interpretation or manuscript writing; and there were minor differences in the intervention intensity in cohorts 1–2 vs 3–5 (although there were no statistically significant differences between cohorts in virtually all outcomes).

In conclusion, a WFPB lifestyle intervention with moderate exercise was more effective than SMC at improving glycaemic control, body weight, waist circumference and inflammation. It also lessened the need for glucose-lowering and cardiovascular medications and induced type 2 diabetes remission in some participants. Overall, our findings support the ‘food as medicine’ concept and suggest that WFPB interventions with moderate exercise may dramatically reduce the risk of comorbidities. A WFPB diet with moderate exercise can be offered as a highly effective, evidence-based lifestyle intervention for individuals with type 2 diabetes.

## Supplementary Information

Below is the link to the electronic supplementary material.

ESM (PDF 229 KB)

## Data Availability

The datasets generated during and/or analysed in the current study are available from the corresponding author upon reasonable request.

## Abbreviations

DBP: Diastolic blood pressure
DiRECT: Diabetes Remission Clinical Trial
hsCRP: High-sensitivity C-reactive protein
MES: Medication effect score
PB+Ex: Whole-food, plant-based intervention with moderate exercise
RMI: Republic of the Marshall Islands
SBP: Systolic blood pressure
SMC: Standard medical care
VLCD: Very-low-calorie diet
WFPB: Whole-food, plant-based
