# Correction: A whole-food, plant-based intensive lifestyle intervention improves glycaemic control and reduces medications in individuals with type 2 diabetes: a randomised controlled trial

**DOI:** 10.1007/s00125-024-06332-z

**Published:** 2024-11-25

**Authors:** Cody J. Hanick, Courtney M. Peterson, Brenda C. Davis, Joan Sabaté, John H. Kelly

**Affiliations:** 1 Department of Nutrition Sciences, University of Alabama at Birmingham, Birmingham, AL USA; 2 Brenda Davis Nutrition Consultation Services, Kelowna, BC Canada; 3 School of Public Health, Center for Nutrition, Lifestyle, and Disease Prevention, Loma Linda University, Loma Linda, CA USA; 4 Department of Preventive Medicine, School of Medicine, Loma Linda University, Loma Linda, CA USA; 5 Lifestyle Health Education Inc., Rocky Mount, VA USA

**Correction: Diabetologia**

10.1007/s00125-024-06272-8

The contributions of Canvasback Missions, Inc.; Jamie. W Spence; Jacqueline L. Spence; and Hak Jae Chung were not fully detailed in the Acknowledgements. Additionally, in the Funding section, details for the U.S. Army Medical Research Acquisition Activity and Canvasback Missions, Inc. were not complete. The original article has been corrected.

