# Supplementary material for: A whole-food, plant-based intensive lifestyle intervention improves glycaemic control and reduces medications in individuals with type 2 diabetes: a randomised controlled trial
Source: Diabetologia. 2024 Sep 21;68(2):308–19. doi: 10.1007/s00125-024-06272-8 (PMC11732952; doi:10.1007/s00125-024-06272-8)

ONLINE-ONLY SUPPLEMENTAL MATERIALS

Additional Information on Methods

For a full detailed description of the protocol, see the protocol manuscript by Davis et al. (2019).

Cultural Adaptation. The study team worked alongside the RMI Ministry of Health when designing the intervention to be culturally sensitive. Staff were provided with cultural sensitivity training, and indigenous Marshallese staff were hired to perform as many study functions as possible, including meal preparation and group sessions. Study documents, lectures, and instructions were provided in the Marshallese language, and Marshallese cuisine recipes were incorporated into the intervention. Classes were taught primarily by J.H.K. and B.C.D., with some classes taught by Marshallese medical professionals or by visiting experts in lifestyle medicine. Finally, group exercises were led by a Marshallese staff member familiar to the community for leading similar activities.

Representativeness of the Study Population. The study was conducted from June 2006 to December 2008. During year 1, participants were selected from the general population with the help of local Marshallese leaders. During year 2, participants were additionally recruited through the RMI Ministry of Health's Diabetes Wellness Clinic. To the best of our knowledge, the study population is representative of those living in the RMI with type 2 diabetes. However, due to exclusion of participants with evidence of more severe cardiovascular disease and of participants with an HbA_{1c} <8.0% who were not on antihyperglycemic medications, our study population may be slightly more or less healthy than the overall RMI population with type 2 diabetes.

Positionality Statement. Four of five co-authors are U.S. citizens, and one is a Canadian citizen. We recognize that our experiences related to North American culture, traditions, and norms—especially regarding food, human health, and healthcare—may influence our contributions to this paper. All human beings have cultural and societal norms that produce biases which we cannot avoid or change, but only acknowledge and seek to consciously minimize their effects. However, we are glad to say that the funding for the study and the motive of the research team were to help correct injustices done to the Marshallese people in the past.

ESM Table 1—Educational group classes for the PB+Ex group.

Diet and Diabetes Class Topics	Exercise Class Topics
What is diabetes? Definition and classifications. What causes diabetes? Insulin resistance, prediabetes risk and factors, and metabolic syndrome.	Why exercise?
Clinical signs and symptoms, complications, and diagnosis of diabetes. Stages of high blood sugar. Is diabetes reversible? Treatment of diabetes and restoring insulin sensitivity.	Exercise and inactivity
Lifestyle medicine and diabetes. Lifestyle and diabetes graduates share experiences.	Interval training #1
Link between diet and diabetes. Obesity and diabetes. The Diabetes Wellness Program diet and food classes	Interval training #2
Diet & diabetes lecture #1: The most healthful foods and dietary components	Muscle and fat loss
Diet & diabetes lecture #2: The most harmful foods and dietary components	Exercise and aging
Diabetes lab tests and blood sugar monitoring. Lifestyle and diabetes program graduates share experiences.	Exercise guidelines
Bringing the program home and taking personal responsibility for health (brand new car comparison)	Why exercise?
The Diabetes Wellness Program dietary and lifestyle principles	
Stress management and diabetes	

ESM Table 2—Cardiometabolic risk factors. Shown below are the effects of a whole-food, plant-based diet with moderate exercise (PB+Ex) and standard medical care (SMC) on cardiometabolic risk factors. Week 0 values represent baseline values and are shown as raw mean ± SD, while week 2-24 values represent changes relative to baseline and are shown as least squares mean ± SE.

	**SMC** (n=70)	**PB+Ex** (n=66)	**Between-Group Difference**	***p***
**HbA_{1c}, mmol/mol**				
Week 0	92 ± 23	88 ± 23		
Week 2	-6 ± 3	-11 ± 3	-4 ± 4	0.34
Week 6	-7 ± 2	-20 ± 2	-13 ± 3	<0.0001*
Week 12	-7 ± 2	-22 ± 2	-14 ± 3	<0.0001*
Week 24	-8 ± 2	-16 ± 2	-8 ± 3	0.01*
**HbA_{1c}, %**				
Week 0	10.5 ± 2.1	10.2 ± 2.1		
Week 2	-0.6 ± 0.3	-1.0 ± 0.3	-0.4 ± 0.4	0.33
Week 6	-0.6 ± 0.2	-1.8 ± 0.2	-1.2 ± 0.3	<0.0001*
Week 12	-0.7 ± 0.2	-2.0 ± 0.2	-1.3 ± 0.3	<0.0001*
Week 24	-0.7 ± 0.2	-1.4 ± 0.2	-0.7 ± 0.3	0.02*
**HbA_{1c}+MES, mmol/mol**				
Week 0	98 ± 23	98 ± 26		
Week 2	-7 ± 4	-10 ± 4	-3 ± 6	0.61
Week 6	-8 ± 2	-21 ± 3	-13 ± 4	0.0004*
Week 12	-7 ± 2	-26 ± 3	-19 ± 4	<0.0001*
Week 24	-7 ± 2	-18 ± 3	-11 ± 4	0.005*
**HbA_{1c}+MES, %**				
Week 0	11.1 ± 2.1	11.1 ± 2.3		
Week 2	-0.7 ± 0.4	-0.9 ± 0.3	-0.3 ± 0.5	0.58
Week 6	-0.8 ± 0.2	-1.9 ± 0.2	-1.2 ± 0.3	0.0004*
Week 12	-0.6 ± 0.2	-2.4 ± 0.2	-1.7 ± 0.3	<0.0001*
Week 24	-0.6 ± 0.2	-1.6 ± 0.3	-1.0 ± 0.3	0.005*
**Glucose, mmol/l**				
Week 0	13.1 ± 4.3	12.7 ± 4.1		
Week 2	-0.7 ± 0.4	-4.0 ± 0.4	-3.2 ± 0.6	<0.0001*
Week 6	-0.6 ± 0.4	-3.6 ± 0.4	-3.0 ± 0.6	<0.0001*
Week 12	-0.4 ± 0.4	-2.5 ± 0.4	-2.1 ± 0.6	0.0007*
Week 24	-0.7 ± 0.4	-1.4 ± 0.5	-0.8 ± 0.6	0.24
**Insulin, pmol/l**				
Week 0	58.0 ± 45.5	63.4 ± 54.2		
Week 2	0.0 ± 5.2	-10.1 ± 5.2	-10.1 ± 7.4	0.17
Week 6	4.8 ± 5.3	-12.6 ± 5.4	-17.4 ± 7.6	0.02*
Week 12	-1.9 ± 4.9	-11.3 ± 5.2	-9.5 ± 7.2	0.19
Week 24	-4.8 ± 5.6	-10.0 ± 6.7	-5.2 ± 8.7	0.55

**HOMA-IR**				
Week 0	5.61 ± 6.31	5.64 ± 4.54		
Week 2	-0.78 ± 0.61	-2.52 ± 0.56	-1.74 ± 0.83	0.04*
Week 6	0.44 ± 0.58	-2.54 ± 0.59	-2.98 ± 0.83	0.0004*
Week 12	-0.36 ± 0.53	-2.10 ± 0.56	-1.74 ± 0.77	0.02*
Week 24	-0.27 ± 0.61	-0.91 ± 0.82	-0.64 ± 1.03	0.53
**MES, mmol/mol**				
Week 0	10 ± 8	13 ± 11		
Week 2	-1 ± 1	-5 ± 1	-5 ± 1	0.002*
Week 6	0 ± 1	-5 ± 1	-5 ± 2	0.002*
Week 12	0 ± 1	-5 ± 1	-6 ± 1	0.0002*
Week 24	2 ± 1	-5 ± 1	-8 ± 2	<0.0001*
**MES, %**				
Week 0	0.9 ± 0.7	1.2 ± 1.0		
Week 2	-0.1 ± 0.1	-0.5 ± 0.1	-0.4 ± 0.1	0.002*
Week 6	0.0 ± 0.1	-0.5 ± 0.1	-0.4 ± 0.1	0.002*
Week 12	0.0 ± 0.1	-0.5 ± 0.1	-0.5 ± 0.1	0.0002*
Week 24	0.2 ± 0.1	-0.5 ± 0.1	-0.7 ± 0.1	<0.0001*
**Weight, kg**				
Week 0	76.2 ± 14.6	79.0 ± 13.7		
Week 2	-0.1 ± 0.3	-1.5 ± 0.3	-1.4 ± 0.4	0.001*
Week 6	-0.3 ± 0.3	-2.8 ± 0.3	-2.6 ± 0.4	<0.0001*
Week 12	-0.4 ± 0.3	-2.9 ± 0.3	-2.5 ± 0.5	<0.0001*
Week 24	0.2 ± 0.3	-2.5 ± 0.3	-2.7 ± 0.5	<0.0001*
**Waist circumference, cm**				
Week 0	96.6 ± 10.0	98.8 ± 10.0		
Week 2	-1.3 ± 0.6	-2.1 ± 0.6	-0.8 ± 0.9	0.34
Week 6	-0.9 ± 0.6	-2.7 ± 0.7	-1.8 ± 0.9	0.04*
Week 12	-1.3 ± 0.7	-3.2 ± 0.7	-1.9 ± 0.9	0.04*
Week 24	2.0 ± 0.7	-1.8 ± 0.7	-3.8 ± 1.0	0.0002*
**Total cholesterol, mmol/l**				
Week 0	4.88 ± 0.97	4.88 ± 1.16		
Week 2	-0.05 ± 0.11	-0.53 ± 0.10	-0.47 ± 0.15	0.001*
Week 6	0.03 ± 0.11	-0.35 ± 0.10	-0.38 ± 0.15	0.01*
Week 12	-0.03 ± 0.12	-0.19 ± 0.12	-0.16 ± 0.17	0.34
Week 24	-0.24 ± 0.12	-0.09 ± 0.13	0.15 ± 0.17	0.39
**Triglycerides, mmol/l**				
Week 0	2.04 ± 1.09	2.18 ± 1.14		
Week 2	-0.06 ± 0.09	-0.53 ± 0.09	-0.47 ± 0.12	0.0002*
Week 6	-0.08 ± 0.09	-0.36 ± 0.09	-0.28 ± 0.12	0.02*
Week 12	-0.04 ± 0.09	-0.43 ± 0.09	-0.38 ± 0.13	0.003*
Week 24	0.00 ± 0.09	-0.23 ± 0.10	-0.22 ± 0.13	0.09
**LDL cholesterol, mmol/l**				
Week 0	3.03 ± 0.91	3.01 ± 1.04		
Week 2	0.05 ± 0.13	-0.08 ± 0.11	-0.13 ± 0.17	0.43

Week 6	0.12 ± 0.11	-0.11 ± 0.11	-0.23 ± 0.16	0.13
Week 12	0.18 ± 0.12	0.08 ± 0.13	-0.10 ± 0.18	0.58
Week 24	-0.13 ± 0.14	0.01 ± 0.14	0.14 ± 0.19	0.47
**HDL cholesterol, mmol/l**				
Week 0	0.91 ± 0.21	0.94 ± 0.26		
Week 2	-0.04 ± 0.02	-0.09 ± 0.02	-0.05 ± 0.03	0.09
Week 6	-0.04 ± 0.02	-0.08 ± 0.02	-0.04 ± 0.03	0.18
Week 12	-0.07 ± 0.02	-0.09 ± 0.02	-0.02 ± 0.03	0.57
Week 24	0.00 ± 0.02	-0.02 ± 0.02	-0.01 ± 0.03	0.64
**Systolic blood pressure, mmHg**				
Week 0	118 ± 21	125 ± 22		
Week 2	2 ± 2	-6 ± 2	-8 ± 3	0.02*
Week 6	-4 ± 2	-11 ± 3	-8 ± 3	0.03*
Week 12	-2 ± 3	-5 ± 2	-3 ± 4	0.42
Week 24	-1 ± 3	-7 ± 3	-6 ± 4	0.13
**Diastolic blood pressure, mmHg**				
Week 0	72 ± 11	74 ± 10		
Week 2	1 ± 1	-4 ± 1	-5 ± 2	0.003*
Week 6	-2 ± 1	-6 ± 1	-4 ± 2	0.03*
Week 12	0 ± 1	-3 ± 1	-3 ± 2	0.08
Week 24	-1 ± 1	-4 ± 1	-2 ± 2	0.28
**Heart rate, beats/min**				
Week 0	78 ± 9	78 ± 11		
Week 2	3 ± 1	0 ± 1	-3 ± 2	0.11
Week 6	0 ± 1	-4 ± 1	-4 ± 2	0.02*
Week 12	0 ± 1	-5 ± 1	-5 ± 2	0.02*
Week 24	-1 ± 1	-4 ± 1	-3 ± 2	0.10
**hsCRP, nmol/l**				
Week 0	29 ± 19	34 ± 22		
Week 2	-3 ± 3	-16 ± 3	-14 ± 4	0.0003*
Week 6	0 ± 3	-15 ± 3	-14 ± 4	0.0003*
Week 12	-4 ± 3	-12 ± 3	-9 ± 4	0.02*
Week 24	1 ± 3	-10 ± 3	-11 ± 4	0.005*

MES=Medication Effect Score, which represents the total dosage of antihyperglycemic medications. HOMA-IR=homeostatic model assessment for insulin resistance. hsCRP=high-sensitivity C-reactive protein. * p<0.05

ESM Fig. 1—Participant flow diagram.

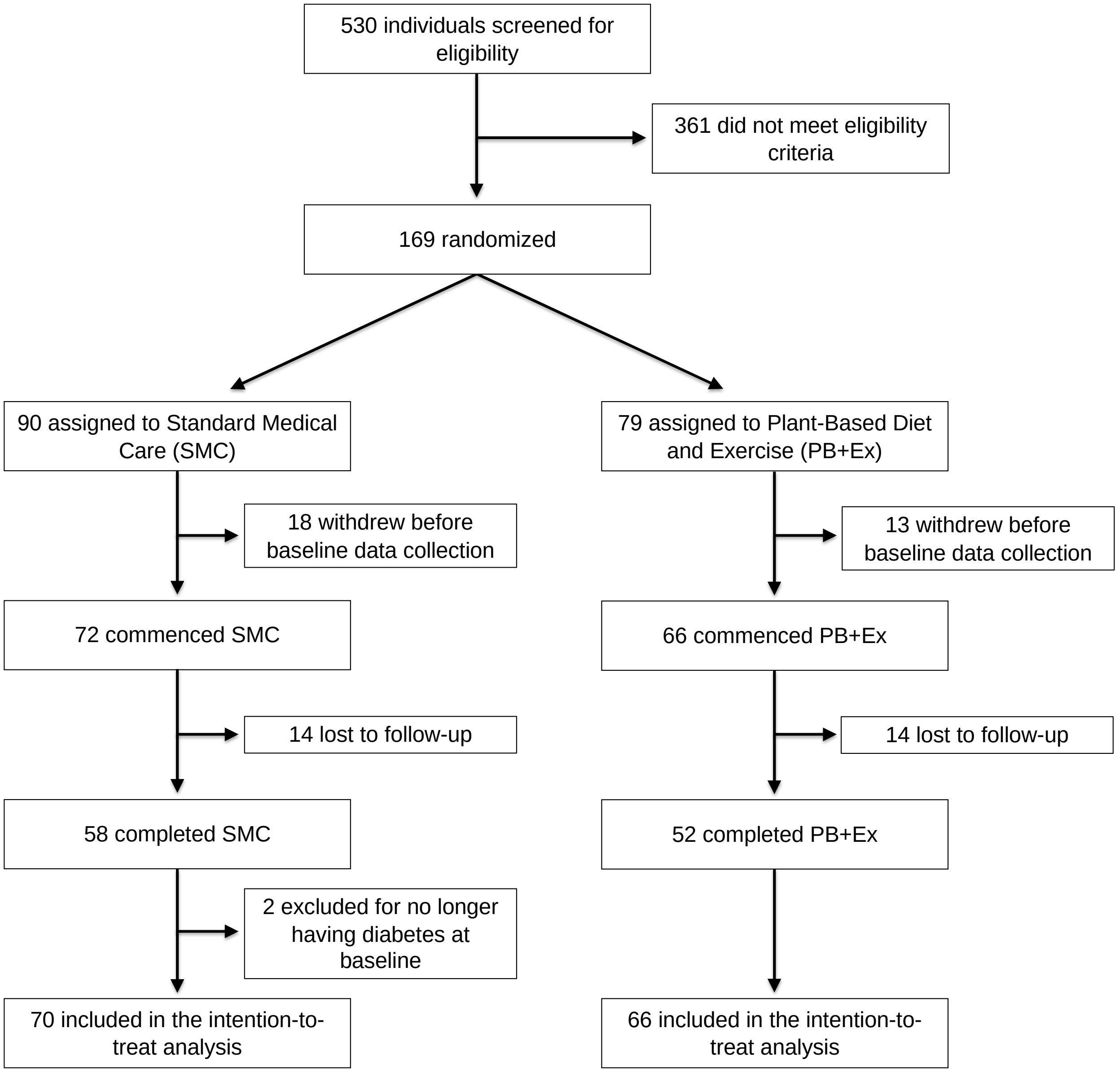

ESM Fig. 2—Additional cardiometabolic endpoints. Relative to standard medical care (SMC), a whole-food, plant-based diet with moderate exercise (PB+Ex) decreased the (A) total dosage of diabetes medications as measured by the medication effect score (MES) at all timepoints, (B) homeostatic model assessment for insulin resistance (HOMA-IR) at all timepoints except week 24, and (C) waist circumference at all timepoints except week 2. There were no differences in (D) high-density lipoprotein (HDL) cholesterol at any timepoint. Data shown are least-squares means ± SEs. * $p<0.05$

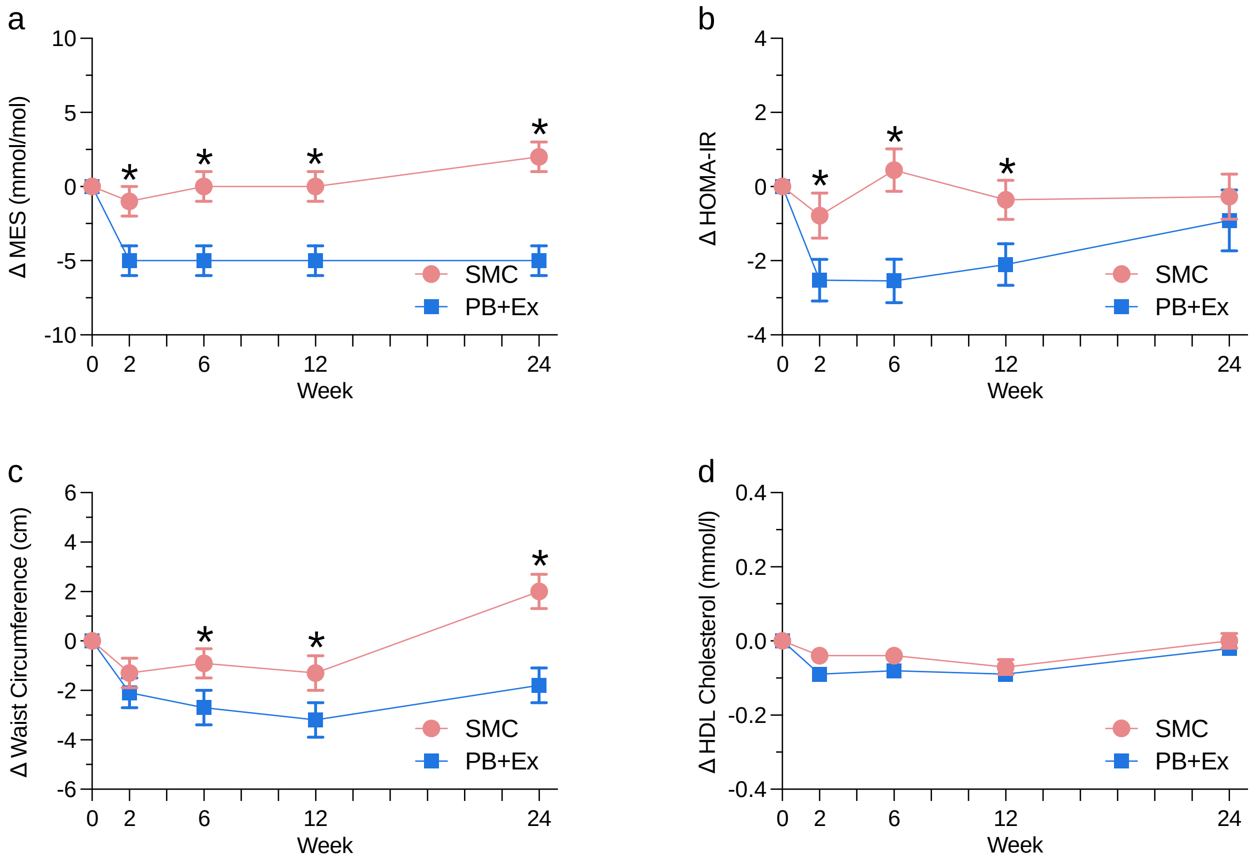

Supplement: Supplementary file 1 — ESM (PDF 229 KB) [file 125_2024_6272_MOESM1_ESM.pdf]
